# Supplementary material for: Yiai Fuzheng decoction inhibits triple-negative breast cancer by remodeling the immune microenvironment
Source: Front Immunol. 2025 Sep 30;16:1615631. doi: 10.3389/fimmu.2025.1615631 (PMC12518410; doi:10.3389/fimmu.2025.1615631)
Supplement: Supplementary file 2 [file DataSheet1.zip › Supplementary File 2/Treatment/C_vs_D_vs_H/06_Pathway_Analysis/Pathway_Analysis/Sig_Pathways_Name/Starch and sucrose metabolism.pdf]

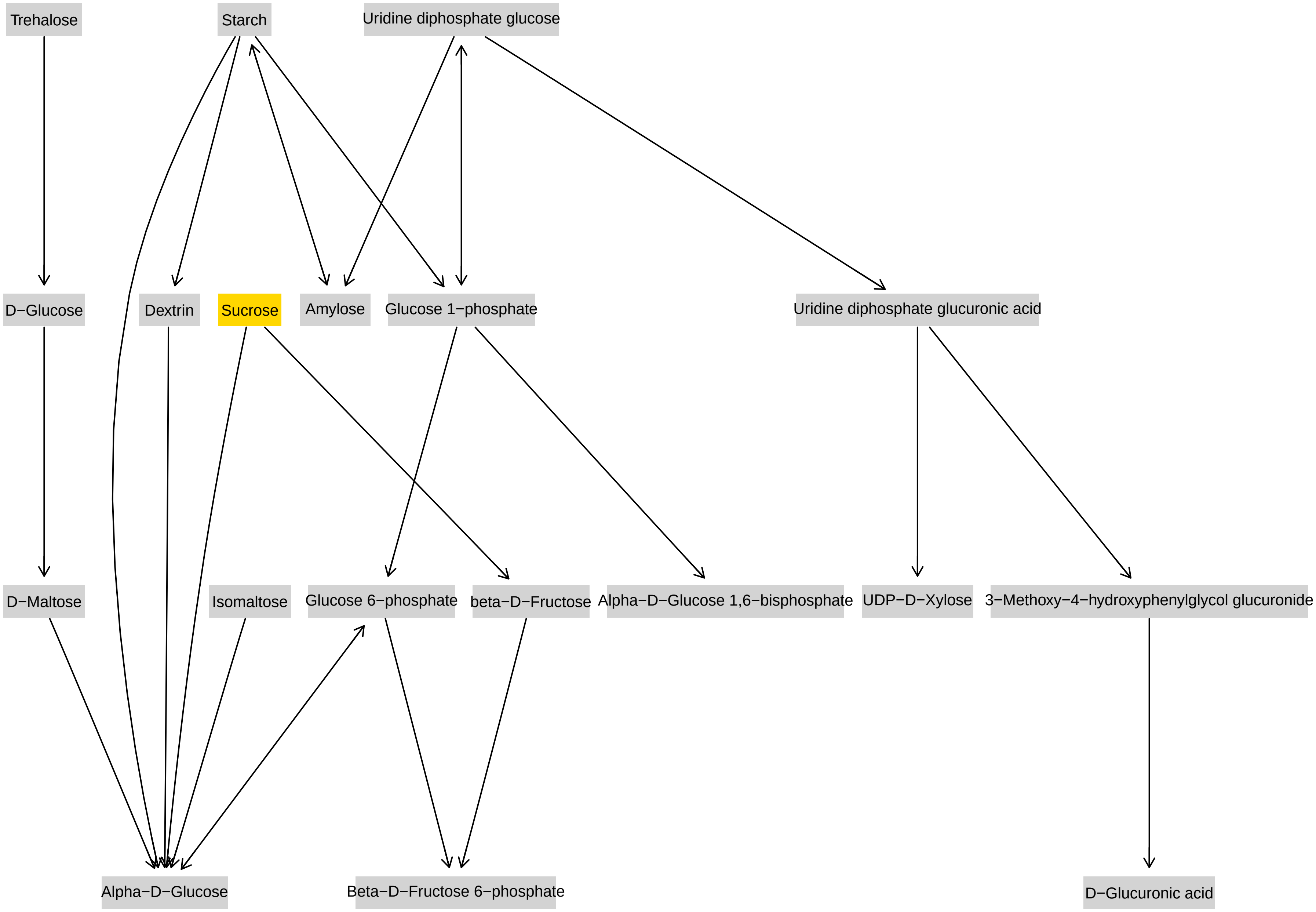
Trehalose
Starch
Uridine diphosphate glucose
D−Glucose
Dextrin
Sucrose
Amylose
Glucose 1−phosphate
Uridine diphosphate glucuronic acid
D−Maltose
Isomaltose
Glucose 6−phosphate
beta−D−Fructose
Alpha−D−Glucose 1,6−bisphosphate
UDP−D−Xylose
3−Methoxy−4−hydroxyphenylglycol glucuronide
Alpha−D−Glucose
Beta−D−Fructose 6−phosphate
D−Glucuronic acid
